# The usefulness of vertebral needle targeting simulation training system using ray-summation imaging: experimental study

**DOI:** 10.1007/s11604-022-01291-0

**Published:** 2022-06-10

**Authors:** Fumiya Uchiyama, Tomoyuki Noguchi, Shunsuke Kamei, Koji Yamashita, Yoshitaka Shida, Takashi Okafuji, Ryotaro Kamei, Tsuyoshi Tajima

**Affiliations:** 1 Department of Radiology, Japanese Red Cross Medical Center, 4-1-22 Hiroo, Shibuya-ku, Tokyo, 150-8935 Japan; 2 Department of Radiology, National Hospital Organization Kyushu Medical Center, 1-8-1 Jigyohama, Chuo-ku, Fukuoka, Fukuoka 810-8563 Japan; 3 Department of Clinical Research, National Hospital Organization Kyushu Medical Center, 1-8-1 Jigyohama, Chuo-ku, Fukuoka, Fukuoka 810-8563 Japan; 4 Department of Radiology, Center Hospital, Center for Clinical Sciences, National Center for Global Health and Medicine, 1-21-1 Toyama, Shinjuku-ku, Tokyo, 162-8655 Japan; 5 Education and Training Office, Department of Clinical Research, Center for Clinical Sciences, National Center for Global Health and Medicine, 1-21-1 Toyama, Shinjuku-ku, Tokyo, 162-8655 Japan; 6 Department of Radiology, Tokai University Hachioji Hospital, 1838 Ishikawa-machi, Hachioji, Tokyo 192-0032 Japan

**Keywords:** Vertebroplasty, Vertebral needle targeting, Learning curve, Virtual fluoroscopy, Ray-summation image

## Abstract

**Purpose:**

Using the multi-detector computed tomography and related three-dimensional imaging technology, we developed a vertebral needle targeting simulation training system named spinal needling intervention practice using ray-summation imaging (SNIPURS). Herein, we assessed the utility of SNIPURS by evaluating changes in the learning curves of SNIPURS trainees.

**Methods:**

Twenty-one examinees were enrolled: seven experienced operators (expert group), seven trainees with coaching (coaching group), and seven trainees without coaching (non-coaching group). They performed six tests of vertebral needle targeting simulation on the workstation-generated spinal ray-summation images of six patients with vertebral fractures. In each test, they determined the bilateral trans-pedicular puncture points and angles on two thoracic and two lumbar vertebrae on ray-summation imaging (i.e., 8 simulations per test). The coaching group received coaching by a trainer after Tests 1 and 4, while the others did not. Scores were given based on the trans-pedicular pathway (1 point) or not (0 point). Eight virtual needles were evaluated in each of Tests 1–6.

**Results:**

Among the three groups, the expert group had the highest average scores on Tests 1–4 (expert: 3.86, 6.57, 7.43, and 7.57; coaching: 1.86, 6.14, 6, and 6.29; and non-coaching: 1.14, 4.14, 4.71, and 4.86). The coaching group’s scores caught up with the expert groups’ average scores on Tests 5 and 6, whereas those of the non-coaching group did not (expert and coaching: 7.86 and 8.00, non-coaching: 5.86 and 7.14). All examinees in the expert and coaching groups achieved a perfect score on the final Test 6, whereas three of the seven non-coaching trainees did not.

**Conclusion:**

SNIPURS might be suitable for vertebral needle targeting training. The coaching provided during SNIPURS training helped the trainees to acquire the spinal puncture techniques in PVP.

## Introduction

Vertebral needle insertion is an important step in the percutaneous vertebroplasty (PVP) procedure. The common puncture route for PVP procedures is trans-pedicular from the dorsal region with the patient in the prone position [1]. The procedure with this route is inherently safe, because there are no other adjacent anatomical structures that can be damaged by the needle as long as its position is carefully selected. A dorsal puncture also allows compression hemostasis of a patient in the supine position after a PVP procedure to minimize postoperative bleeding. Punctures deviating from the trans-vertebral route can cause neurological damage or hemorrhagic complications [2–6], and an improper puncture can lead to an improper distribution of cement, that is, cement leakage to the outside of the vertebrae, which might result in serious complications [6–9].

Vertebral needle insertion is not always easily accomplished, as it is difficult to recognize and puncture the narrow pedicle of the vertebral arch in fluoroscopy [10–12]. Radiology trainees must learn the basic procedures and be given opportunities for intensive practice, although the available training period is limited and there are concerns about patient safety [13]. A new method of puncture training for beginners based on a new concept has thus been desired.

Recent developments in multi-detector computed tomography (MDCT) and its associated three-dimensional CT imaging (3D-CT) technology have contributed considerably to interventional radiology [14–16]. The authors in previous papers pointed out that the use of 3D-CT would give a first-hand understanding of the entire structure being evaluated, and that the location details could be identified by examining the original axial image. Ray-summation imaging, which is also known as a “virtual fluoroscopy imaging”, shows a projected image that is similar to digital radiography [17]. A ray-summation image is created from the signal values of the ray with the average value in the projection and is used in practice for the pre-procedural planning of percutaneous trans-hepatic biliary drainage and bronchoscopy for pulmonary peripheral lesions [15, 16]. These facts led us to the hypothesis that the ray-summation imaging could be applied to the puncture simulation training in PVP. We thus developed a vertebral needle targeting training system named spinal needling intervention practice using ray-summation imaging generated from MDCT data (SNIPURS).

Radiology trainees can undergo simulation training for the vertebral needle targeting on the SNIPURS system, using the workstation-generated spinal ray-summation images. Sufficient preoperative simulation training can improve the trainees’ skills without compromising patients’ safety and reduce the risk to patients undergoing PVP. We conducted the present study to assess the usefullness of the SNIPURS system as a preparatory training for PVP by evaluating changes in trainees’ learning curve.

## Methods

### Study design

This study was approved by the Ethics Review Committee of our hospital (approval no. NCGM-G-003447-00). Written informed consent was obtained from all of the examinees. Specifically, examinees decided to participate in this study on their own will after receiving an explanation of the content of this study and an explanation that they would not suffer unreasonable disadvantages by refusing to participate this study. The need for written informed consent from the patients whose anonymous MDCT data were used for the SNIPURS testing was waived.

### A spinal ray-summation image of the patients

We first selected the patients whose MDCT data were used for the testing as follows. PVP was performed in 199 consecutive patients including 213 procedures in our hospital during the period from April 2014 to December 2017. Of those patients, we selected the thoraco-lumbar spine CT data of six patients based on the following criteria: (1) The target vertebra for the vertebral needle targeting simulation training test is limited to the 11th thoracic to 3rd lumbar vertebrae, where vertebral fractures were frequently encountered and treated with PVP [18]; (2) Genant classification [19] grade 2 (moderate) or 3 (severe) fractures were present in both the lumbar and thoracic vertebrae of a candidate patient; (3) Genant classification grade 1 (mild) fractures or grade 0 (unfractured) vertebra were present in both the lumbar and thoracic vertebrae. We included criterion (3) because most trainees were inexperienced in PVP and would benefit from training with a simple vertebra as well as deformed vertebrae for their training in vertebral needle targeting.

The characteristics of the selected patients and target vertebra were summarized in Table 1. The patients’ preoperative 1-mm thickness MDCT images were transferred to a workstation (Synapse Vincent^®^ software, Fujifilm Co., Tokyo, Japan) to generate the spinal ray-summation images.

**Table 1:** The characteristics of the selected patients and target vertebra

Test no.	Test1	Test2	Test3	Test4	Test5	Test6
Level of target vertebra (Grading by Genant classification [17])	T11(3)	T11(0)	T11(0)	T11(3)	T11(0)	T11(0)
	T12(0)	T12(2)	T12(3)	T12(0)	T12(3)	T12(3)
	L2(1)	L2(3)	L2(0)	L1(3)	L2(2)	L2(0)
	L3(3)	L3(0)	L3(3)	L2(0)	L3(0)	L3(2)

*T* thoracic vertebra, *L* Lumbar vertebra

### Examinees

Since we could not find existing studies or tests that are similar to our present investigation, we could not properly estimate the statistical effect size to calculate the minimum sample size of examinees [20]. We thus sought to recruit at least three subjects in each group so that we could perform the Kruskal–Wallis test [21–23]. We enrolled seven experienced examinees (the expert group) and 14 trainees who were alternately assigned to two groups (coaching and non-coaching groups, *n* = 7 each). The members of the expert group were all radiologists, and seven of 14 trainees were medical interns and the others were radiological residents. Table 2 provided the profiles of the examinees in each group.

**Table 2:** The profiles of the examinees for the three groups

Item	Expert group	Coaching group	Non-coaching group
Number of examinee	7	7	7
Radiologist	7		
Radiological resident		3	4
Medical intern		4	3
Age: average years (range)	42 (32–54)	27 (25–32)	28 (25–36)
Sex: M/F	5/2	4/3	6/1
Years of experience as a medical doctor: median (range)	18 (8–30)	3 (1–8)	4 (1–12)
Months of PVP experience: median (range)	55 (24–204)	0 (0–10)	0 (0–10)

### Virtual PVP procedure and score evaluation

A single trainer (F.U.) first explained the SNIPURS procedures to the examinee, including the imaging tools to zoom, rotate, and reset the view on the workstation with the computer mouse, using the spinal ray-summation image of Test 1. The examinee was not allowed to try to complete the pretest examination, e.g., to point out the puncture site or obtain the result before starting the test described below.

**Fig. 1:**
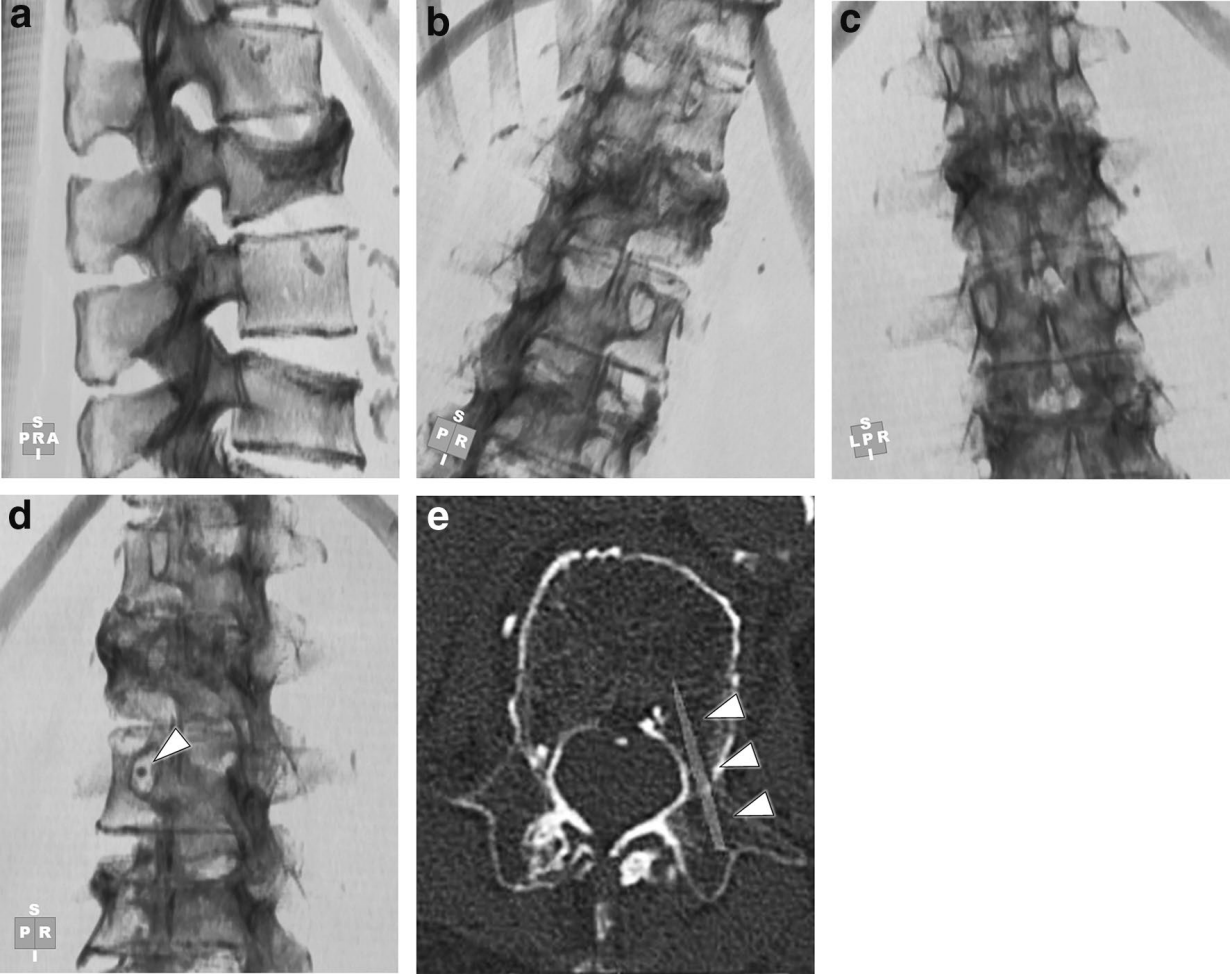
**a** Lateral, **b** right posterior clockwise rotation oblique, **c** posterior clockwise rotation, and **d** left posterior oblique spinal ray-summation, and **e** Multi Planer Reconstruction (MPR) images to schematically illustrate the process of SNIPURS. The examinee freely orients the spinal ray-summation image to view the appearance of the spine in any direction (**a**–**c**) and determines the on-end puncture point in the selected direction (**d**: arrowhead). As the post-processing, the virtual needle (1 mm in diameter and 50 mm in length) is visualized with an excavation tool, and the path of the puncture needle is evaluated on the workstation (**e**: arrowhead)

**Fig. 2:**
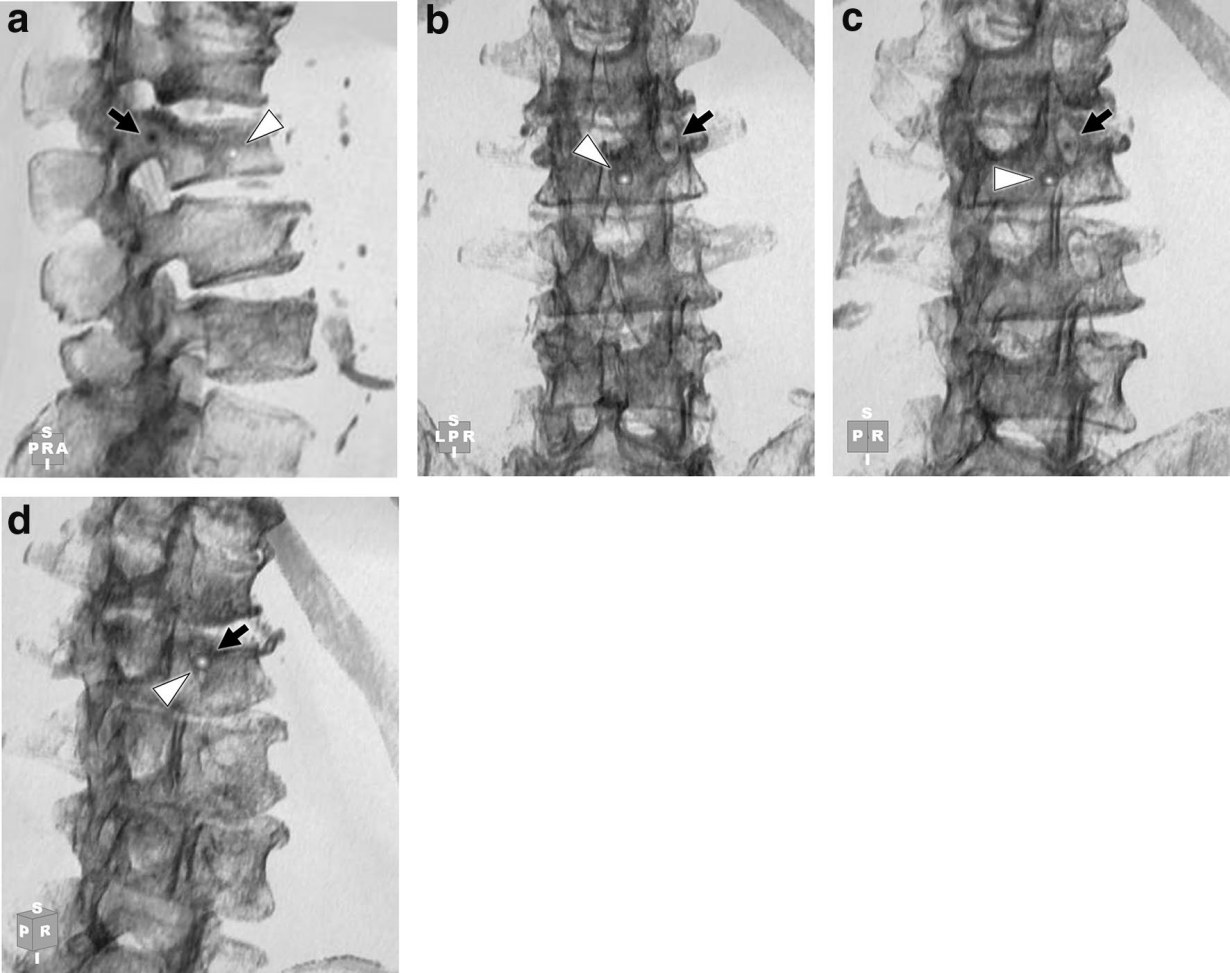
**a** Lateral, **b** posterior, **c**, left posterior oblique, and **d** left posterior elevation oblique spinal ray-summation images to schematically illustrate the coaching provided with SNIPURS in this study. The ideal position of the needle was determined so that the needle could pass through the center of the vertebral arch and the tip of the needle could reach the anterior third of the vertebral body. The trainer places the two dot marks at the center of the pedicle arch (**a**, **b**; black dots with arrowheads) and at the ideal tip of the puncture needle on the ray-summation image (**a**, **b**; white dots with arrows). A trainee in the coaching group freely orients the ray-summation image (**a**–**c**), overlays both marks (**d**; a white dot with an arrowhead and an arrow), and thus understands the optimal puncture direction

The examinee then started the SNIPURS training as follows: (1) The examinee viewed the spinal ray-summation image from any directions using the mouse on the workstation (Fig. 1a–c), (2) selected a single-puncture view to perform puncturing on-end (which is called the bull’s-eye modification method [24]), (3) determined the position of the puncture point on that view (Fig. 1d), and (4) performed steps (1) to (3) to determine the puncture points and angles of the bilateral sides for two thoracic and two lumbar vertebrae with the trans-pedicular approach on the ray-summation images. After the selection of the puncture location, post-processing was conducted: a total of eight virtual needles (1 mm in diameter, 50 mm in length) were visualized with an excavation tool, and then the pathway of the puncturing needle was evaluated on the workstation (Fig. 1e). The examinee was not allowed to obtain the score result while performing SNIPURS.

Scores were given based on whether the puncturing needle’s pathway was inside or overlapped on the bone cortex of the vertebral pedicle (trans-pedicular route, 1 point), or outside the vertebral pedicle (non-trans-pedicular route, 0 points), which was evaluated by the trainer. Each examinee completed a total of six tests (Tests 1–6) with the highest possible score of 8 points on each test, and the learning curves for the average score of each of the three examinee groups was obtained.

### Coaching for technical development

The coaching group received the coaching after Tests 1 and 4 by the trainer using the spinal ray-summation images of Tests 1 and 4. The ideal position of the needle was determined so that the needle could pass through the center of the vertebral arch and the tip of the needle could reach the anterior third of the vertebral body. The coaching is as follows: (1) The trainer put two dot marks in the center of the pedicle arch and in the ideal tip of the puncture needle on the ray-summation image (Fig. 2a). (2) The trainees confirmed the dot marks on the axial 2D-CT, coronal 2D-CT, and ray-summation images. (3) The trainee rotated the ray-summation image until the two marks overlapped (Fig. 2b–d). The trainee was then able to understand which working angle was the best for the puncture.

### Evaluation items and statistical analyses

We evaluated if the examinees accomplished the following three qualifying scores: (1) To determine that SNIPURS depended on the operator’s PVP experience and was suitable for vertebral needle targeting training, the expert group would achieve the highest scores among the three groups, which was assessed using the intergroup comparisons. (2) To determine that the coaching was effective, the individual coaching group should get the post-coaching scores better than the pre-coaching scores and should catch up with the expert group for the scores by the final Tests 6, which was evaluated using the intragroup comparisons. In addition, we performed an intergroup comparison between the coaching group and the non-coaching group because it could be predicted that score would increase step by step without any coaching. (3) We required all three groups of examinees to reach a perfect score by Test 6.

The intergroup comparisons among the three groups for each test and the intragroup comparisons among the six tests of each group were performed using the Kruskal–Wallis test and Scheffe's post hoc test combined with the Bonferroni correction (nine comparisons). The significance level was set to 0.05. The statistical analyses were properly performed using Excel 2013 (Microsoft, Seattle, WA) with the add-in software Statcel-3 [23].

## Results

**Fig. 3:**
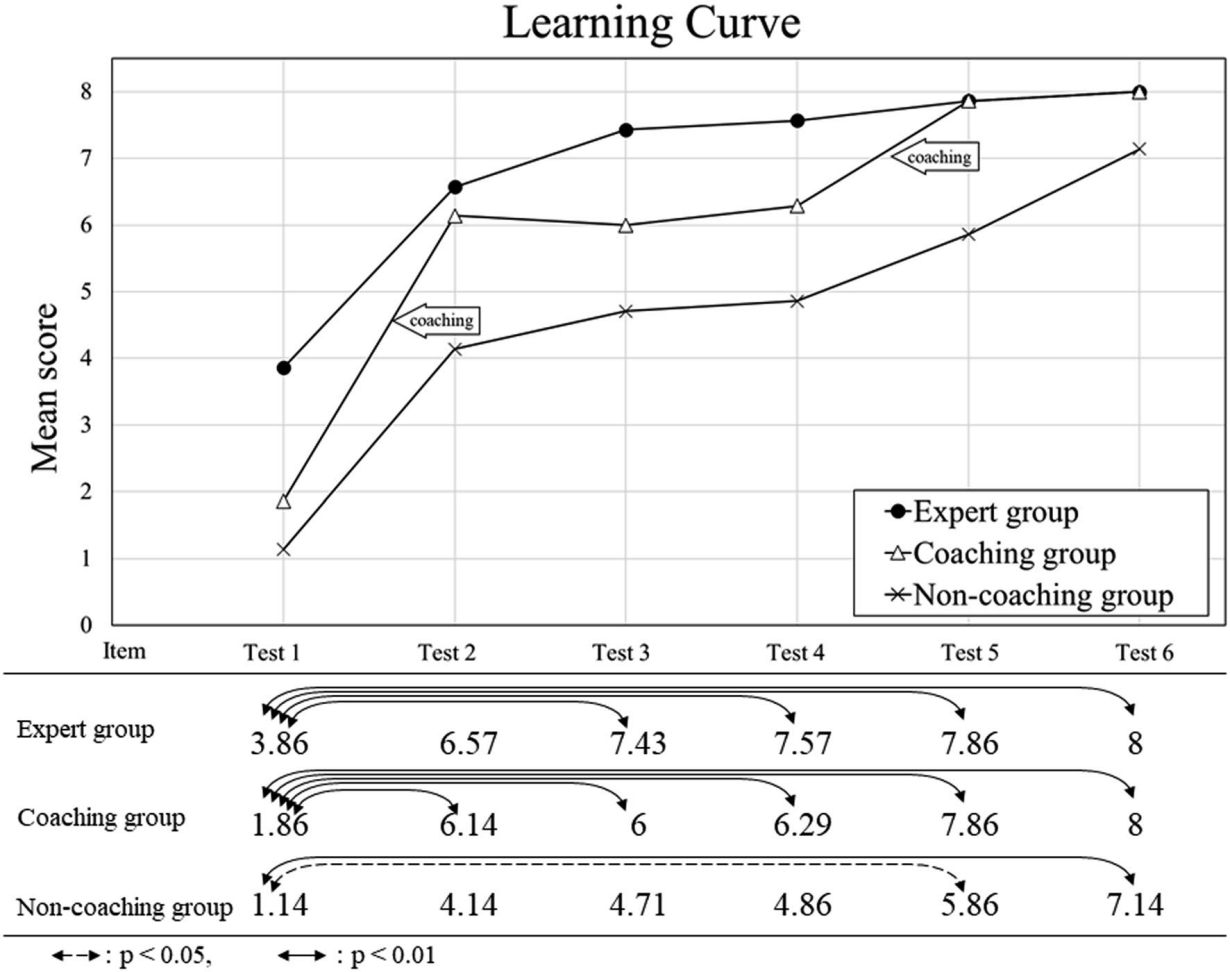
Learning curve of the expert (a line with dot markers), coaching (a line with triangle markers), and non-coaching (a line with X markers) groups. All three groups achieved an increase in the average scores in proportion to the number of tests. The Test 2 scores of the coaching group increased significantly after the first coaching. In the semifinal Test 5 (after the second coaching), the coaching group caught up with the expert group in scores. In the final Test 6, all examinees in the expert and coaching groups achieved perfect scores, but the trainees in the non-coaching group did not

Figure 3 depicts the learning curve of average scores and the results of the intragroup comparisons among the 3 groups. All groups showed an increase in the average scores. Average (median/range) scores for Test 1 to 6 were 3.86 (3/1–7), 6.57 (7/3–8), 7.43 (7/7–8), 7.57 (8/7–8), 7.86 (8/7–8), and 8.00 (8/8–8) in the expert group, 1.86 (2/0–3), 6.14 (7/4–8), 6 (7/3–8), 6.29 (6/4–8), 7.86 (8/7–8), and 8.00 (8/8–8) in coaching group, 1.14 (1/0–3), 4.14 (3/2–8), 4.71 (4/2–8), 4.86 (5/1–7), 5.86 (7/2–8), and 7.14 (8/5–8) in non-coaching group, respectively. On the other hand, the intergroup comparisons for each of the six tests revealed no significant difference.

1. **The expert group had the highest scores among the three groups**
  The expert group had the highest SNIPURS score of the three groups. The intragroup comparisons identified significant differences within the expert group for Test 1 versus (vs.) Test 3 (*p* = 0.002), Test 1 vs. Test 4 (*p* = 0.001), Test 1 vs. Test 5 (*p* < 0.001), and Test 1 vs. Test 6 (*p* < 0.001).
2. **The coaching group had the post-coaching scores better than the pre-coaching scores and caught up with the expert group for the scores by the final Test 6.**
  In the coaching group, the Test 2 scores performed after the first coaching were significantly higher than the Test 1 scores (*p* = 0.001), and their Test 5 scores performed after the second coaching were better than their Test 4 scores and caught up with the expert group’s Test 5 scores. The intragroup comparisons of Test 4 vs. 5 scores within the coaching group did not reveal a significant difference. The intergroup comparisons in Test 2 or Test 5 between the coaching group and the non-coaching group also did not show the significant difference.
3. **The non-coaching group could not reach a perfect score by the final Test 6.**
  All of the examinees in the expert and coaching groups got a perfect score (8 points) on Test 6, whereas only three of the seven trainees in the non-coaching group achieved a perfect score on Test 6. However, significant differences in the non-coaching group were shown by the intragroup comparisons of Tests 1 vs. Test 5 (*p* = 0.045) and Test 1 vs. Test 6 (*p* = 0.002). The average score of the non-coaching examinees never caught up with those of the expert or coaching groups.

## Discussion

3D imaging techniques enable a quick perception of entire structures [14], and the details of various positions can be obtained by examining the original images or multiplanar reconstruction (MPR) images. Kinoshita et al. evaluated the usefulness of virtual fluoroscopy-based pre-procedural planning in the percutaneous trans-hepatic biliary drainage procedure, and they concluded that intraoperative referencing using virtual fluoroscopy imaging shortens the X-ray fluoroscopy time and within-procedure times [15]. An accurate and safe method for performing PVP under 3D radiography guidance generated by cone-beam CT using angiographic equipment has also been reported [11]. These 3D imaging techniques are useful for treating patients, and they can be applied to training as well. Our SNIPURS training procedure may be a useful tool for trainees to learn a procedure on a workstation without risking a patient’s safety.

In the present assessment of the utility of SNIPURS, we emphasized the following points. We speculated that since SNIPURS should be a training system for spinal puncture in PVP, PVP experts with the considerable experience who had no coaching should be able to score higher than other trainees. We also felt that successful trainees must achieve the same score as the experts by the time they completed the SNIPURS training, because a failure to puncture the vertebral body must be avoided in PVP in practice. Therefore, the coaching was preferable for trainees to learn the vertebral needle targeting efficiently. The learning curves of the expert, non-coaching, and coaching groups in this study could be regarded as the reference standard for (i) clinicians who are experienced in performing PVP and take the SNIPURS test, (ii) beginners who need to increase their degree of proficiency, and (iii) beginners whose scores on the SNIPURS test could improve with coaching, respectively. Our results will provide important data when SNIPURS is used as an instructional approach for PVP.

In the expert group, the significant differences were seen in Tests 1 vs. 3, Tests 1 vs. 4, Tests 1 vs. 5, and Tests 1 vs. 6, suggesting that a significant scoring ability for this simulation test can be obtained after just Tests 1 and 2. Ideally, the PVP experts should have achieved high scores on Tests 1 and 2 as well. Low score on those tests in this study may suggest that preliminary experience was still required to become accustomed to SNIPURS even in the expert group. An additional reason for the experts’ initial low scores could be that SNIPURS used the single-puncture view. The puncture direction is determined using a biplane view in most facilities. SNIPURS with a single-puncture view might be difficult for experts who have usually performed vertebral needle targeting with the isocenter puncture (ISOP) method [10], cathelin needle-assisted puncture (CAP) method [25], or the angle fixation method [26]. Nevertheless, the experts obtained the highest scores of the three groups on each test and got a perfect score on Test 6 with no coaching.

The expert group tended to have higher scores than the coaching and non-coaching groups, especially on Test 1, which suggests that the ability of the coaching and non-coaching groups to grasp SNIPURS the first time tended to be lower than that of the expert group. However, the between-group differences did not reach significance, perhaps because the expert group did not acquire the ideal high scores (from the first Test) as mentioned above.

Among the examinees who received coaching, a significant increase in score was observed for Test 2 (after the first coaching). In addition, after the second coaching (post-Test 4), the coaching group's average score on Test 5 caught up with that of the expert group. All of the examinees who received coaching achieved the perfect score of 8 points on Test 6, and thus the learning mission in SNIPURS was completed with two brief coaching sessions.

Among the examinees of the non-coaching group, a significant increase in score was observed for both Test 5 and Test 6 compared to the score on Test 1; that is, no significant learning results were obtained from Tests 1 to 4. This result may indicate that the learning curve in the non-coaching group tended to increase slowly. In addition, there were 3 trainees in the non-coaching group who did not reach the perfect 8-point score on Test 6, suggesting that they might need to take further simulation training.

Based on the results of this study, we reached the conclusion that SNIPURS with two coaching sessions can be recommended to successfully help trainees effectively acquire a precise perception of spinal puncture techniques in PVP. Losch et al. made a similar proposal: that coaching creates a high degree of satisfaction and is superior in helping participants attain their goals, whereas the absence of coaching and other support from a trainer is not sufficient for high goal attainment [27].

There are some limitations to our investigation. The sample size of examinees (*n* = 7, each group) and the test samples were insufficient. Our results also do not fully demonstrate that SNIPURS for learning vertebral needle targeting is actually useful for trainees to master needle puncture techniques. Further research is necessary to confirm and advance our present simulation training study.

In conclusion, we evaluated the vertebral needle targeting simulation training system SNIPURS for clinicians' preparation to perform the PVP procedure. SNIPURS might be suitable for vertebral needle targeting training. The provision of coaching during SNIPURS trainees helped the trainees acquire the spinal puncture techniques used in PVP.

## Abbreviations

3D-CT: Three-dimensional computed tomography
CAP: Cathelin-needle-assisted Puncture
ISOP: Isocenter Puncture
MDCT: Multi-detector computed tomography
MPR: Multi-Planar Reconstruction
PVP: Percutaneous vertebroplasty
SNIPURS: Spinal Needling Intervention Practice Using Ray-Summation imaging
